# 

**Published:** 1857-06

**Authors:** 


### RECEIPTS ON SUBSCRIPTIONS,

UP TO MAY 28TH, 1857.

### ILLINOIS.

| | | |
|---|---|---|
| Dr. S. Vredenburgh, - | Vol. 4&5, | $5.00 |
| “ Geo. W. Brooks, - | “ 4,6&6, | 6.00 |
| “ R. J. Stough, - | “ 5, | 2.00 |
| “ P. Gregg, - - | “ 4&5, | 4.00 |
| Drs. Brackett & Buckly, | “ 4&5, | 4.00 |
| Dr. C. A. Logan, - | “ 4&5, | 4.00 |
| “ Wickeisham, - - | “ 6, | 2.00 |
| “ L. P. Cheeney, - | “ 6, | 2.00 |
| “ Martin, - - - | “ 4&5, | 4.00 |
| “ F. W. Hanee, - | “ 5, | 2.00 |
| “ A. W. Butler, - - | “ 6, | 2.00 |
| “ E. A. Goodwin, - | “ 5, | 2.00 |
| “ L. Clark, - - - | “ 6, | 2.00 |
| “ A. M. Cuttin, - - | “ 4&5, | 4.00 |
| “ C. N. Andrews, - | “ 4&5, | 4.00 |
| “ M. S. McArthur, - | “ 4&5, | 4.00 |
| “ Lyman, - - - | “ 5, | 2,00 |
| “ L. L. Lake, - - | “ 4&5, | 4.00 |
| “ R. S. Maloney, - | “ 5, | 2.00 |
| “ D. McM. Marshall, | “ 6, | 2.00 |
| “ J. H. Wilkinson, - | “ 6, | 2.00 |
| “ R W. Palmer, - | “ 4&5, | 5.00 |
| “ A. M. D. Hughes, | “ 6, | 2.00 |
| “ F. R. Payne, - - | “ 6, | 2.00 |
| “ C. M. Robertson, - | “ 6, | 2.00 |
| “ J. A. Roman, - - | “ 6, | 2.00 |
| “ C. D. Henton, - | “ 6, | 2.00 |
| “ L. M. Dimmick, - | “ 6, | 2.00 |
| “ A. Hager, - - - | “ 6, | 2.00 |
| “ J. Perry, - - | “ 6, | 2.00 |
| “ T. Mead, - - - | “ 4&5, | 4.00 |
| “ E. R. Cruthers, - | “ 4&5, | 4.00 |
| “ J. P. Rogers, - - | “ 4,5&6, | 6.00 |
| “ G. N. Galtre, - - | “ 5&6, | 4.00 |
| [illegible]. Lang, - - - | “ 4,5&6, | $6.00 |
| “ H. R. Lathy, - - | “ 5&6, | 4.00 |
| “ Thos. Stanton, - | “ 5, | 2.00 |
| “ S. T. Trowbridge, | “ 5&6, | 4.00 |
| “ S. Z. Baldwin, - | “ 4&5, | 4.65 |
| “ G. Holmes, - - | “ 6, | 2.00 |
| “ Geo. T. Allen, - | “ 6, | 2.00 |
| Drs. Ross & Gurham, - | “ 6, | 2.00 |
| “ S. M. Meeker, - | “ 6, | 2.00 |
| “ Q. R. Bridges, - | “ 6, | 2.00 |
| “ L. C. White, - - | “ 5&6, | 4.00 |
| “ D. T. Hyner, - - | “ 6, | 2.00 |

### MICHIGAN.

| | | |
|---|---|---|
| Dr. O. C. Otis, - - - | Vol. 6, | 2.00 |
| “ B. Hudson, - - - | “ 4, | 3.00 |
| “ John McLean, - - | “ 5, | 2.00 |

### IOWA.

| | | |
|---|---|---|
| Dr. W. W. Line, - - | Vol. 5, | 2.00 |
| “ Geo. Reeder, - - | “ 6, | 2.00 |
| “ O. George, - - | “ 3&4, | 5.00 |

### INDIANA.

| | | |
|---|---|---|
| Dr. R. D. Marzy, - - | Vol. 6, | 2.00 |
| “ W. H. Bull, - - | “ 6, | 2.00 |
| “ R. C. Moore, - - | “ 4&5, | 4.00 |
| “ L. H. Kennedy, - | “ 4&5, | 4.00 |
| “ G. M. Carpenter, - | “ 6, | 2.00 |
| “ Jno. Lewis, - - | “ 6, | 2.00 |

### WISCONSIN.

| | | |
|---|---|---|
| Dr. D. LaCount, - - | Vol. 5, | 2.00 |
| “ B. F. White, - - | “ 6, | 2.00 |
| “ H. A. Yeomans, - | “ 6, | 2.00 |
| “ A. Young, - - | “ 5&6, | 4.00 |
| “ Jas. Prentice, - | “ 6, | 2.00 |
| “ J. C. Mills, - - | “ 5&6, | 4.00 |

